# A processing-type active real-time traceable certification system

**DOI:** 10.1038/s41598-023-50315-w

**Published:** 2024-01-25

**Authors:** Chia-Chun Wu, Chung-Huei Ling, Min-Shiang Hwang

**Affiliations:** 1 Department of Industrial Engineering and Management, National Quemoy University, Kinmen, 892 Taiwan; 2 Department of Computer Science and Information Engineering, Asia University, Taichung, 41354 Taiwan; 3 Department of Medical Research, China Medical University Hospital, China Medical University, Taichung, 404333 Taiwan

**Keywords:** Computational biology and bioinformatics, Mathematics and computing

## Abstract

In addition to substituting low-price and low-quality materials for high-quality materials at the food processing stage, many dishonest businesses risk adulterating chemical materials in products to reduce production costs or increase product flavor with chemical synthesis spices. As a result, the risks to food safety are increased. Most safety management and certification regulations proceed with on-site examination or sampling inspection. As current certification systems lack complete tracking and real-time certification processes, they cannot comprehensively check foods’ processing and production processes and contents. Hence, food safety problems sway consumers’ trust and confidence in certification systems. This study intends to improve the agricultural processing end’s current food traceability certification system. Adding the design of raw material total quantity control provides a complete and sound real-time certification mechanism for citizens and businesses to assure consumer rights.

## Introduction

To establish an agricultural traceability system, the participants are internationally requested to provide the input sources, the output destinations, and the batch relationship of additives in the processing between these two in the food value chain process^1^. A complete traceable and trackable system is then structured through the traceability information network. For the processing plants participating in the food value chain, the primary function is to use raw materials and additives for the processing and outputs according to the standard model to have the product composition conform to the rules^2,3^. A certification unit must regularly examine the plants and inspect whether the product operation models conform to the regulations and compositions to secure the sales source of products.

The current traceability system is established with zoning control. Five stages are covered from production to consumers: agricultural production, processing, distribution, retail, and consumers. Each stage presents the recorded contents, such as the production, delivery, and other incidental records at the production stage; the stock, manufacturing and processing, and delivery records (containing the signature of the manufacturing responsible person, expiration date, and production line number) at the processing stage; the product stock records, production and processing schedules, delivery, and other records at the distribution stage; and finally the product stock and sales records at the retail stage^4^. By regulating the recorded contents, the information and data of production, handling, processing, distribution, and sales can be reserved and traceable for the entire process to further guarantee food safety from a farm to a dining table^5–7^.

In order to promote the global competitiveness of domestic agriculture and enhance the transformation and upgrade of agriculture, the Council of Agriculture, the current traceability authority, promotes the agriculture and food traceability system geared to international standards, expecting to effectively segment domestically produced agricultural produce from smuggled ones, to create the added value of agricultural produce, and to enhance agriculture in presenting quality, safety, and stable food supply chain advantages^8,9^. However, when food processing technology has been rapidly developing in past years, how to ensure the food safety of food processing products has become the common requirement of global consumers; processing products not in accord with environmental protection and food safety standards will not be easily distributed in international markets.

The use of essence for baked goods, chemical soy sauce, pickled vegetables and fruits with saccharin additive, processing food with genetically modified soybeans, processing meat with ractopamine additive, dehydrated food with preservative, pesticide residue in tea leaves, and adulteration of low-price imported rice in high-price local rice reveal that international certification, which emphasizes on “manufacturing process’ instead of “raw materials,” is insufficient to ensure the food safety. Accordingly, how to ensure the information correctness concerned researchers.

There are four advantages of the proposed traceable certification system:

1. The design of this study assists food processors in establishing a complete food production, sales tracking, and tracing system with low costs through information equipment and functions.
2. The entire management and control with the real-time effect could be achieved through the cloud data system and the auditing processes related to raw material total quantity control to thoroughly solve the information accuracy problem.
3. The certification approach conforms to international trends and public expectations.
4. Planning and establishing a fair third-party certification system could promote consumers’ confidence in products with traceability and allow consumers to enjoy the safety and quality of agricultural products.

The design of this study assists food processors in establishing a complete food production, sales tracking, and tracing system with low costs through information equipment and functions^10–12^.

The following sections are summarized below. The operation model of the current agriculture and food traceability system and the deficiency at the processing stage are described in Section "A current traceability system and assessment structure". The processing-type active tracking system model in this study, the relative data processes, and the total quantity control certification mechanism are explained in Section "Processing-type active traceability tracking system". The differences between the current system and this study are analyzed and compared in Section "Analysis and comparison". Finally, conclusions are proposed in Section "Conclusions and future works".

## A current traceability system and assessment structure

### A current agricultural processing traceability system

**Figure 1:**
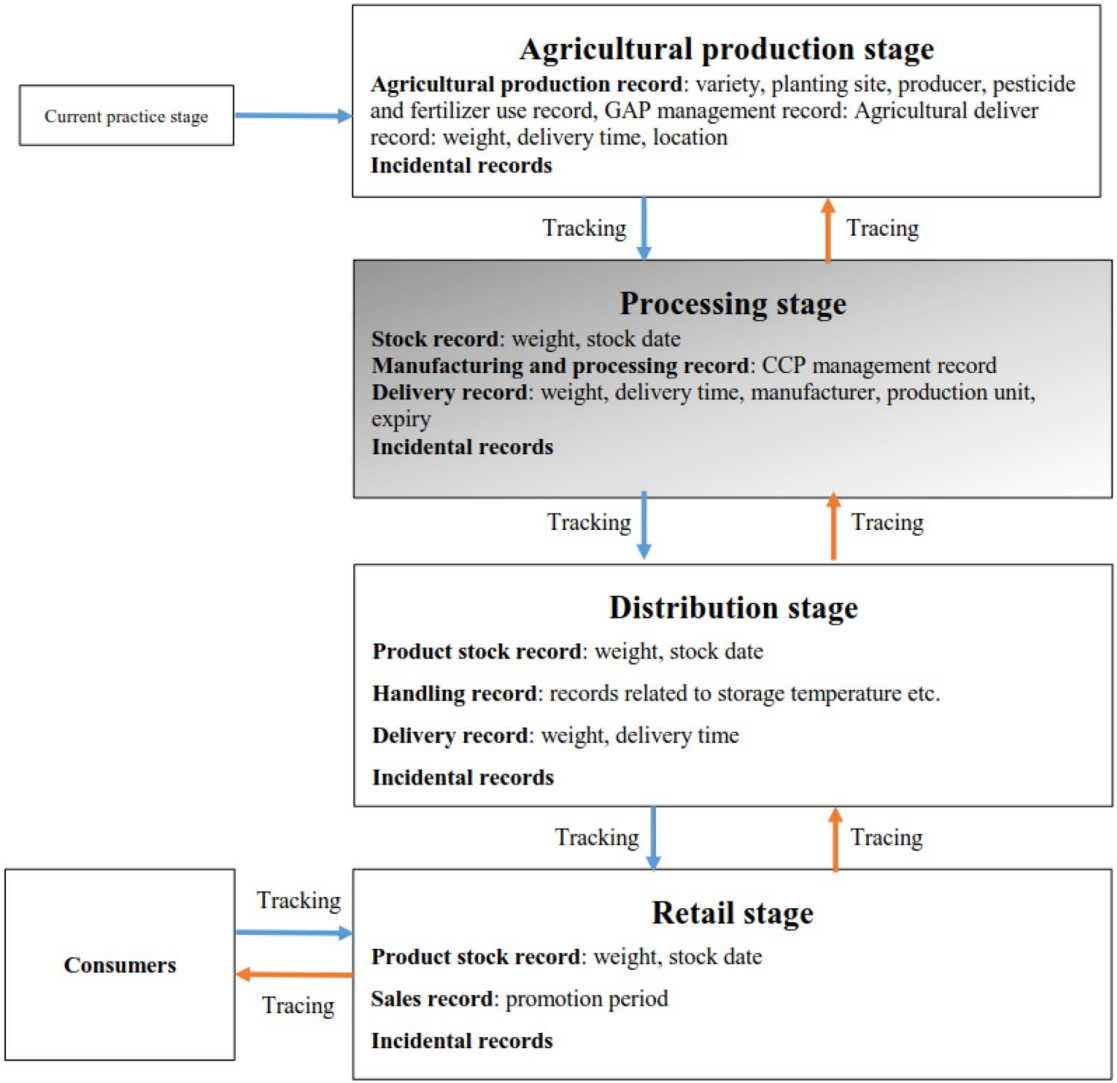
The current traceability system.

Current agricultural processing and production are managed according to the Agricultural Production Certification Act. The open and traceable agricultural records from production, processing, package, and distribution to sales are completed based on the Agricultural and Food Traceability System, i.e., to construct an assurance system with open, transparent, and traceable agricultural traceability information from “farms” to “dining tables” (Fig. 1).

Since the package and quantity of raw materials would be changed at the processing stage, the point is to confirm the correct use and addition of raw materials to prevent adulteration. Accordingly, the agricultural traceability information from production and processing to sales could be traced; that is, the comprehensive source control from a farm to a dining table, including the sources of soil and water, the agricultural material safety, the production management in the midstream, the quality, sanitation, safety of agricultural produce in the downstream. It is similar to the identity of agricultural produce for consumers to understand all information in the agricultural supply and marketing processes through the open and traceable information, so it guarantees the purchased agricultural produce is safe, assured, and reliable, and it creates three wins among producers (farmers), consumers, and distributors.

### Assessment of current processing plants

Aiming at raw materials and processing processes in processing plants, the current traceability assessment structure merely precedes documentary examination and random sampling inspection. Being restricted to workforce and objects, the certification authority is unable to visit the production site of every factory for the assessment of confirm the raw materials to the inspection standards and the production process to the food processing standards in a processing plant by doing sampling inspection of the products, and strictly review the records of each batch of goods under the monitoring or the certification authority. It focuses on the documentary review of the processing process and the production conforming to the operating standards and then irregularly examines or inspects each part of the process afterward. Anti-forgery and adulteration are often discovered afterward or never found out. The assessment structure shows that the current system cannot certify the authenticity of product data in real time. Although the data are complete, the authenticity and continuity cannot be confirmed, so forgery, modification, and adulteration cannot be avoided. Malpractice generated in the operation process in a processing plant cannot be timely mastered, but merely the relevant products are recycled and destroyed after the problems burst out. Therefore, the food safety threat to consumers has happened already.

### Defect of current processing plant certification

The current management system of food processing plants still has management and control problems, so food safety problems keep occurring under the operating mechanism. The defects of the current mechanism are briefly explained as follows:

- *Insufficient defense mechanism:* A manufacturer stocks raw materials from a supplier, but he cannot judge the legality and security of such raw materials from the labels. The loophole in the raw material management mechanism results in counterfeit products or the adulteration of industrial raw materials in food raw materials. The insufficient production and sales control of artificial additives or chemical compositions causing illegal or improper additives cannot be effectively monitored and managed.
- *Lack of monitoring mechanism and internal fraud:* Undertaking the products from the production stage, where the complex changes of products (appearance and quantity) result from raw materials and additives, the management and control mechanism at the processing stage are preceded by sampling inspection so that internal fraud in a processing plant cannot be avoided.
- *Lack of instantaneity:* In the product processing process, a manufacturer’s fraud behaviors, which are not immediately discovered, result in the uncertain truth of products.

The food processing plants are encountering a great crisis as the current monitoring and management of food processing are restricted to the system, human resources, and objects. The deceit of unscrupulous manufacturers encumbers reputable manufacturers to be affirmed. It seriously affects the import and export of the food industry. A trackable management mechanism is proposed in this study to overcome the blind spots and problems of the current food processing plant management.

The problem of food adulteration, which has been ongoing for many years, directly impacts the safety of consumers in healthy. Currently, the detection of pollutants is based on physicochemical and instrumental tests. However, progress in counterfeiting is outpacing methods of identifying it. Therefore, we propose a systemic solution to prevent food adulteration at every stage of its production.

## Processing-type active traceability tracking system

A traceability system^13^ designed by the third-party certification attempts to solve the accuracy problem of traceability in this study. Since it presents the effects of fully managing and controlling real-time certification, it is called an active traceability tracking system. Two modules are divided based on regions to cope with different traceability routes. The core technology of a traceability route combines a product variable and a product route variable into a set of single and non-repeated variables. A producer must inform the certification center^14^ of the product number information and the delivery location during the delivery. The certification center precedes the auditing with the relevant information according to the idea of unchangeable product weight and the total weight of the products under this technology. As a result, the dynamic auditing and the real-time certification effects could be achieved to make consumers show greater confidence in the products applying this technology. Table 1 lists the symbols and explanations used in this study for the demonstration.

**Table 1:** Symbol explanation.

Symbol	Explanation
*ID*	Product number QR code
*F*	Wholesaler number $F_1, F_2, \cdots , F_n$
*CF*	Processing plant number $CF_1, CF_2, \cdots , CF_n$
*M*	Retailer number $M_1, M_2, \cdots , M_n$
*TW*	Total weight
$W(\cdot )$	Processing function
*WS*	Processing product electronic label
*FS*	Electronic label $S_1, S_2, \cdots , S_n$
$FS'$	New electronic label $S_1', S_2', \cdots , S_n'$

**Figure 2:**
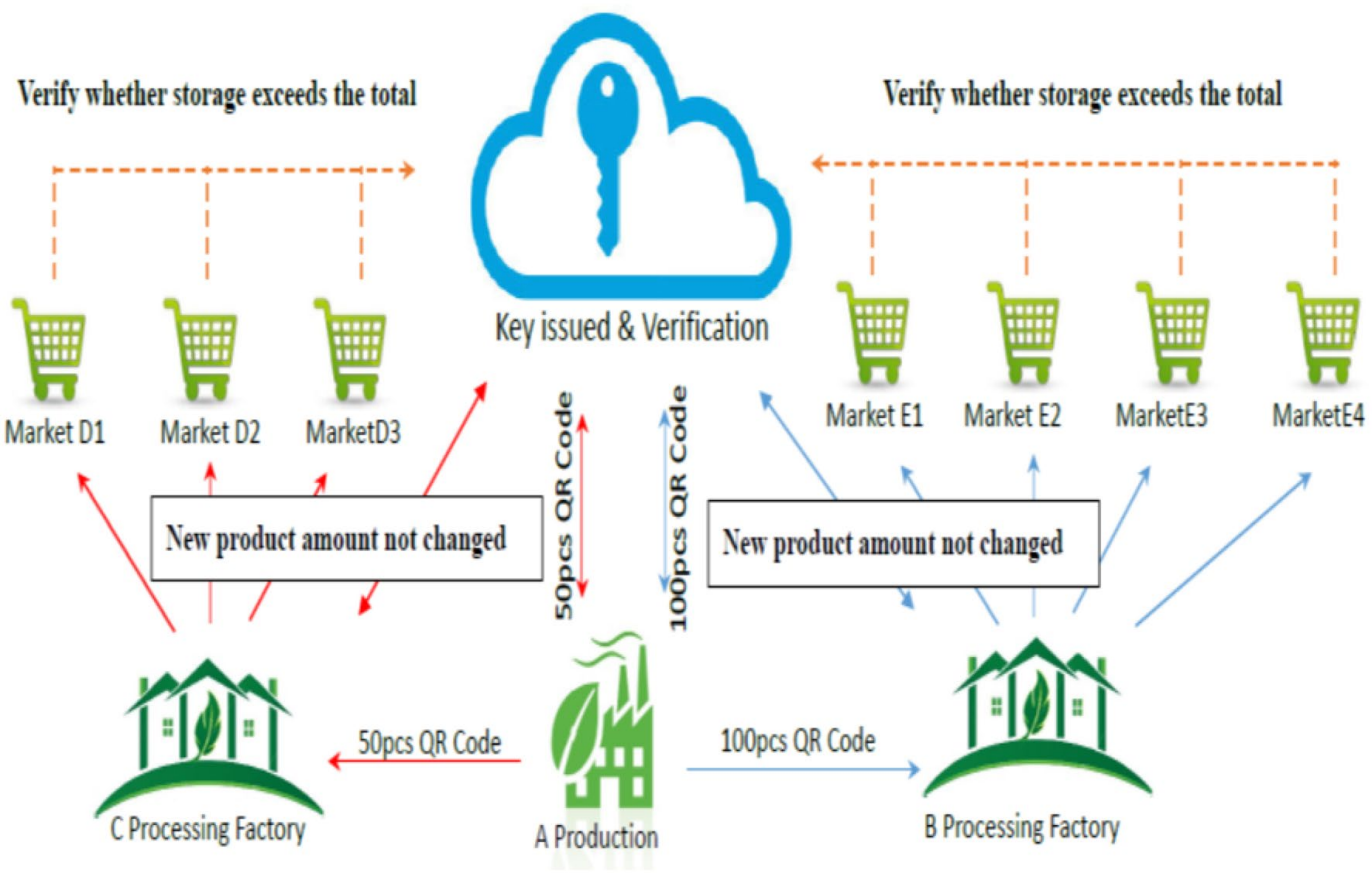
Structure of processing-type active traceability certification tracking system.

The processing-type traceability route shows production $\rightarrow$ processing $\rightarrow$ retail $\rightarrow$ consumer, and the certification system structure is shown in Fig. 2. The cloud in the middle stands for the safety protection mechanism, responsible for the issue and the certification of a traceability route^15^. Products with different total quantities delivered from the production end to a processing plant for processing new products, which are further delivered to sales fields and sold to consumers, are presented on both sides. A processing plant is regarded as a new production factory that has to upload product data for certification^16^. Meanwhile, the total quantity of all raw materials unchanged is also certified.

**Figure 3:**
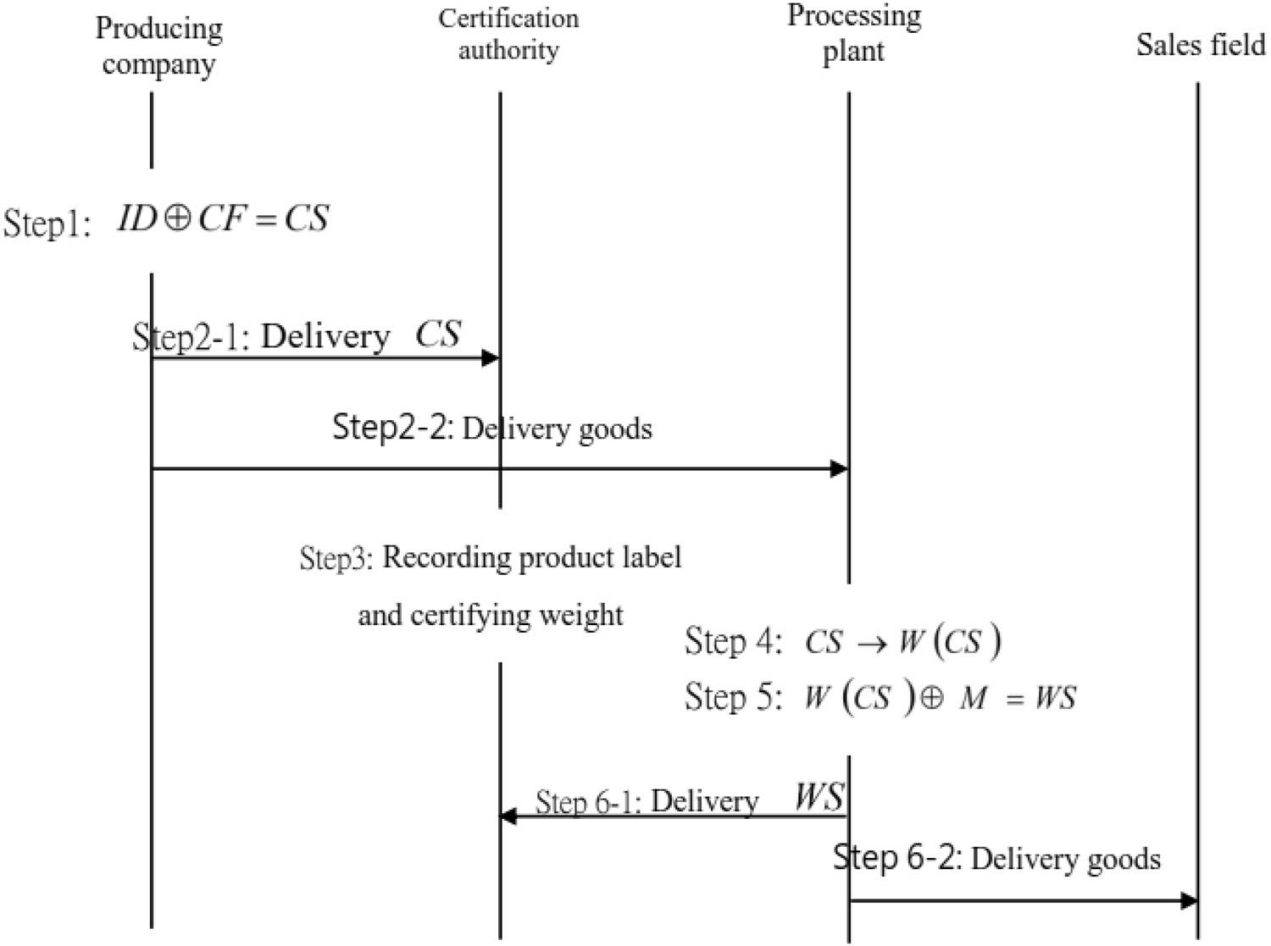
Certification process for processing-type active traceability certification tracking system.

Figure 3 demonstrates the processing-type certification processes.

- *Step 1:* A production factory A combines the delivering information $ID=\{ID_1$, $ID_2$, $\cdots$, $ID_n\}$ with processing plants B and C, $CF=\{CF_1, CF_2, \cdots , CF_n\}$ into the label $CS=\{CS_1, CS_2, \cdots , CS_n\}$, as Equation (1):
  1 $$\begin{aligned} CS_i = ID_i \oplus CF_i, \quad i=1, 2, \cdots , n. \end{aligned}$$
- *Step 2:* The label S is sent to the certification unit, and the products are delivered to a processing plant.
- *Step 3:* The certification unit records the product label *CS*.
- *Step 4:* The processing plant *CF* has to proceed the processing process $W(\cdot )$ on the product *CS* to become a processing product $W(CS_i)$, $i=1, 2, \cdots , n$.
- *Step 5:* The certification unit certifies the raw material *ID* and the product weight *CS*, as Equation (2).
  2 $$ TW(\sum _{i=1}^n ID_i)\phantom{l}_{\ge}^? TW(\sum _{i=1}^n CS_i). $$
- *Step 6:* The processing plant *CF* delivers the processing product $W(CS_i)$ to various sales fields $M_i$ and combines it into a label $WS_i$, as Equation (3).
  3 $$ WS_i = W(CS_i) \oplus M_i, \quad i=1, 2, \cdots , n. $$
- *Step 7:* The processing plant delivers $WS_i$ to the certification unit to record the product label and delivers the processing products to sales fields.

The application of module 2 is demonstrated in the following. Assume that there are 800 raw materials *ID* of a processing product, 100 spices A, and 100 spices B to produce 1000 processing products *WS*, as

$$\begin{aligned} (800 ID) + (100 A) + (100 B) = 1000 WS. \end{aligned}$$

When 200 additives C are added, raw material *ID*, spice A, and spice B remain the same instead; the yield of the processing products *WS* will increase, as

$$\begin{aligned} (800 ID) + (100 A) + (100 B) + (200 C) = 1200 WS. \end{aligned}$$

When additive C is added, and the proportion of raw material *ID* is reduced, spices A and B are not changed; instead, the same raw material could produce more processing products *WS*. Therefore, the total quantity of the processing product *WS* will increase.

$$\begin{aligned} (400 ID) + (100 A) + (100 B) + (400 C)= & {} 1000 WS.\\ (200 ID) + (50 A) + (50 B) + (200 C)= & {} 500 WS. \end{aligned}$$

800 *ID* could produce 1500 processing products *WS*.

In this case, when the proportion of raw material *ID* is reduced, or other additives are added, the total quantity of the processing products *WS* will be changed. With the processing processes in this module, the products would reveal changes in appearance and weight, so the traceability becomes more complicated. This module features the management and control with the total quantity control models, so internal fraud between a processing plant and a wholesaler becomes impossible. Meanwhile, the cloud database system is applied to this system, so the integrity of the system can be certified online and in real-time.

## Analysis and comparison

Table 2 explains the comparison between this system model and the current traceability system model.

**Table 2:** Comparisons between this study and the current traceability model.

Differentiation	Current system model	System model in this study
Defense mechanism: Tractability	Conventional	Automatic
Defense mechanism: Traceability	Conventional	Automatic
Anti-forgery ability: Internal fraud	Low efficacy	High efficacy
Real-time certification ability	No	Yes
Total quantity control	No	Yes

- *Tractability:* Tracking the food traceability system aims to construct the relationship from the traceability source to the downstream. The current system has certain recording standards for products from production, processing, and distribution to retail stages^16^. Such standards cover the production and management records at the production stage, the stock, manufacturing, and processing, and delivery records at the processing stage, the product stock, handling, and delivery records at the distribution stage, and the product stock and sales data at the final retail stage^17^. Moreover, incidental records correspond to product characteristics at each stage for system tracking. ‘Conventional’ in Table 2 refers to the start of tracking reliance on the human operation of the certification unit. Consequently, unlike the system model in this study, the certification unit could immediately construct complete tracking information after receiving the labels transmitted by processing plants, which will be sent to the certification mechanism proceeding to compare the tracking results automatically.
- *Traceability:* Concerning the defense mechanism of trackability, traceability is the reverse engineering of traceability, mainly to construct the relationship from the traceability downstream to the source. The recorded information for tracing is identical for trackability on the standard and the execution^18^. Consequently, the same results are acquired when comparing the current system with the model in this study. Conventional approaches proceed with tracing with references, while the system parameters of the automatic mechanism would develop the tracing function during data certification and examination.
- *Anti-forgery ability:* Internal fraud at the processing stage indicates substituting and adding harmful raw materials for the normal situation. It differs entirely from the external counterfeit label problem at the sales stage. Simply speaking, internal fraud is the behavior of evil processing plants. Since the internal anti-forgery ability^19^ of the current system merely relies on on-site examination, sampling inspection of product stock, and the authenticity of raw materials between manufacturing, processing, and delivery, it cannot comprehensively prevent fraud; it is not an ideal strategy. Under the active structure and matching with the traceability route coding model and total quantity control, this system could detect fraud immediately and deal with it. Compared with the current system, the high efficacy is apparent, and the system could deter bad manufacturers and achieve the demand for traceability safety.
- *Real-time certification ability:* Food safety is a significant issue in people’s livelihood. The authority develops food certification and promotes traceability to control consumers’ safety. Real-time certification refers to the ability to examine products of illegal traceability in real-time. Without the assistance of an information system, it will be too late to examine the factories or inspect the products in the market simply depending on limited human resources. Even though problematic products are inspected, the damaged consumers will find it difficult to make consumers confident in the system. This is why the current system is questioned. Based on the principle of unchangeable total quantity, a processing plant’s stock, delivery, and processing states could be mastered through the active cloud mechanism to ensure the products being examined at any stage in real-time. In this case, the distribution and sales of fraud and counterfeit products could be avoided, so the real-time certification ability of this system will present the practicability to complement and improve the current system.
- *Total quantity control:* Although the current system has a waste report mechanism, the mixed filling and packaging control is relatively loose^14^. Fraud, such as substituting and adding bad raw materials, is difficult to prevent for analyzing and comparing anti-forgery ability. The total quantity control of raw materials in this system could reinforce the loss of the current system to achieve the demand for food safety and satisfy consumers’ expectations.

The following are the advantages of using a cloud system in the proposed system:

1. To enhance the security of the certificated system to prevent food data forgery and food adulteration at every stage of its production.
2. The integrity of the proposed certificated system can be certified online and in real-time.
3. To ensure the products are being examined at any stage in real-time. The distribution and sales of fraud and counterfeit products could be avoided.

## Conclusions and future works

To overcome the blind spots and problems in the certification and management of food processing plants, the processing-type active tracking system proposed in this study utilizes the core technology of traceability routes and the design of raw material total quantity auditing to construct the dynamic auditing and real-time certification effects. Therefore, it complements the loss of the current traceability and reduces the possibility of internal fraud between a processing plant and a wholesaler. In the food value chain, all participants requested to provide the input source and the output destination internationally will reduce the low cost and prevent the forgery of processing plants. More importantly, the design of the real-time certification ability of this system model is thoroughly changed from the currently passive structure. From the analysis and comparison results, the defense mechanisms of internal fraud’s trackability, traceability, and anti-forgery ability present significant contributions^20^. It is believed that the secure system, which can be easily practiced, could assist the authority in solving food processing plants’ defects effectively, further perfect consumer rights protection, and recover consumers’ confidence. Furthermore, most current anti-forgery models focus on trademark or label encryption, so the increasing cost for labels results in farmers’ burden against the promotion, revealing the doubt of fraud from manufacturers. This system shows a better tracing method than the current traceability system to avoid the problems in the trademark and label encryption and achieve the traceability examination under the current model without increasing extra costs.

Meanwhile, this system could keep the instant information and present complete data for all relevant users. The significant effect is that the businesses are willing to provide certification to enhance brand visibility and consumer trust actively. When purchasing products, there are relevant channels for consumers to enquire to ensure consumers’ rights and recover consumers confidence. It is considered a secure system and easily practiced.

With the rapid development of 5G and IoT (Internet of Things), wireless and mobile communication networks are now necessary. All entities in the production chain are sensors, and it is feasible to issue and store electronic certificates. The proposed system is theoretical. There are two works in the future as follows:

1. Applying the proposed system to test based on the actual production process of any selected food product.
2. The synronized problem of the data in the cloud system. How to action once indicated in the event of failure, loss, reduction in the efficiency of the technological process or its increase?

## Data Availability

We clarify that plant materials were not used in the study. The datasets used and/or analysed during the current study available from the corresponding author on reasonable request.
